# Diagnostic performance of multiple ultrasonic modalities for prostate cancer

**DOI:** 10.1016/j.clinsp.2025.100680

**Published:** 2025-05-11

**Authors:** Yushan Liu, Shi Zeng, Dan Zhou, Haiqing He, Shuiqing Wu, Changkun Huang, Kai Ai, Xuan Zhu, Ran Xu

**Affiliations:** a Department of Ultrasound, the Second Xiangya Hospital of Central South University, Changsha, PR China; b Department of Urology, the Second Xiangya Hospital of Central South University, Changsha, PR China

**Keywords:** Gray-scale ultrasound, Doppler ultrasound, Real-time tissue elastography, Contrast-Enhanced Ultrasound, Prostate Cancer

## Abstract

**Objective:**

To evaluate the performance of Grayscale Ultrasound (GSU), Color Doppler Ultrasound/Power Doppler Ultrasound (CDU/PDU), Transrectal Real-Time Tissue Elastography (TRTE) and Contrast-Enhanced Ultrasound (CEUS) in the diagnosis of Prostate Cancer (PCa).

**Method:**

All analyzed patients underwent GSU, CDU/PDU, TRTE and CEUS examinations before radical prostatectomy. Receiver Operating Characteristic (ROC) curves were drawn, and the Areas Under the Curve (AUCs) were calculated for the four ultrasonic modalities alone and in combination. Binary logistic analysis was used to evaluate the predictive value of lesion features observed with the 4 modalities.

**Results:**

The results of the chi-square test showed that the sensitivity, specificity, positive predictive value, negative predictive value, and accuracy rate of TRTE for diagnosing PCa were higher than those of CEUS (p-values were < 0.05 for all). The ROC curves analysis showed that the diagnostic performance of TRTE for PCa was better than that of CEUS (*p* = 0.046). Moreover, the diagnostic performance of the combination of four ultrasonic technologies (AUC = 0.873) was significantly better than that of any technology alone. Multivariate binary logistic regression analysis showed that enhancement strength on CEUS, presence of a blue area on TRTE and total serum Prostate Specific Antigen (PSA) level were independent predictors for prostate malignancy.

**Conclusions:**

The diagnostic performance of TRTE was higher than that of CEUS. Early hyperenhancement on CEUS and stiffness on TRTE had high predictive value for the diagnosis of PCa. The combination of multiple ultrasonic modalities can significantly improve the positive diagnostic rate for PCa over that of a single ultrasonic mode.

## Highlights

• Real-time tissue elastography has excellent diagnostic performance in prostate cancer.
• A blue area on elastography has significant diagnostic value for prostate cancer.
• Multiparametric ultrasound can accurately diagnose prostate cancer.

## Introduction

Prostate Cancer (PCa) is the most common genitourinary system tumor in middle-aged and elderly men, and it is common in most Northern and Western countries. With the “westernization” of lifestyles, the rapid aging of the population and the development of metabolic syndrome, the incidence and mortality of PCa in the studied country have gradually increased in recent years.^1^ PCa is also age-dependent, with a low incidence before the age of 55 and an increasing incidence after that. The onset of PCa is insidious and lacks typical clinical manifestations. Most patients are already in the middle and late stages when they are diagnosed. Therefore, the early clinical diagnosis and treatment of PCa are of great significance in improving the survival rate of patients and their quality of life. At present, early diagnostic tests for PCa mainly include Digital Rectal Examination (DRE), serum Prostate Specific Antigen (PSA) and Magnetic Resonance Imaging (MRI). Elevated PSA levels and abnormal DRE are often clinically considered biopsy indications. However, the PSA of 1/3 of PCa cases can be in the normal range. Only 26 % of men with PSA levels in the “gray area” (4 to 10 ng/mL) were diagnosed with PCa.^2^ Benign prostatic hyperplasia, prostatitis and other non-cancerous lesions can also lead to elevated PSA levels. DRE is limited to palpation of the posterior area of the prostate, which can cause physical discomfort, rectal bleeding and even syncope. Currently, the transrectal, ultrasonographically guided, 12-core systematic biopsy is the commonly used method for the initial diagnosis and grading of prostate cancer.^3^ Transrectal Ultrasound (TRUS) biopsy is the mainstream method for detecting early PCa in clinical practice.^4^ Although MRI or PET-CT (Positron Emission Tomography/Computed Tomography) or even PET-MRI guided puncture is available today, these methods are only available in very few centers. Considering the issue of economic cost, ultrasound is currently the mainstream method for prostate cancer early detection in clinics due to its advantages of low economic cost and universal application in hospitals at all levels. And a good ultrasonic machine does not perform worse than a conventional MRI. The primary concern associated with transrectal prostate biopsy is that it can cause generalized infection or septicemia.

Research on imaging PCa has focused on two platforms: MRI and Ultrasound (US). The American Urology Association and European Association of Urology (EAU) currently recommend the use of multiparameter MRI (mpMRI) and mpMRI-guided prostate biopsies in order to improve the efficacy of systematic ultrasound-guided prostate biopsies for suspicious patients.^5^ Several studies have shown that mpMRI represents the gold standard for the diagnosis of clinically significant Prostate Cancer (csPCa).^6^^,^^7^ Moreover, research by Zhang J et al. showed that targeted MRI/transrectal ultrasound fusion prostate biopsy is superior to systematic prostate biopsy in the detection rate of PCa and csPCa among 161 patients with PI-RADS ≥ 3 (*p* = 0.032).^8^ However, this method is not appropriate for some patients with claustrophobia, prosthetic implants, or renal failure. MpMRI has several limitations including availability, expensive cost, the difficulty of real-time imaging, and low inter-reader agreement. Smaller, low-grade, multifocal, nonindex tumors are more likely to be missed by mpMRI.^9^

US is highly cost-effective and has wide applicability and strong practicability. The main ultrasonic techniques currently used for the diagnosis of PCa include conventional Grayscale Ultrasound (GSU), color Doppler Ultrasound/Power Doppler Ultrasound (CDU/PDU), Ultrasound Elastography (UE), and Contrast-Enhanced Ultrasound (CEUS). GSU shows the anatomical location of the prostate lesion.^10^ CDU/PDU shows the blood flow in the larger hyperplastic vessels in the lesion.^11^ TRTE shows the hardness of the lesion tissue to infer properties about its nature.^12^ CEUS shows new microvessels in the lesion.^13^ This article aims to compare the diagnostic performances of GSU, CDU/PDU, Transrectal Real-Time Tissue Elastography (TRTE), and CEUS in diagnosing PCa, and propose a popularized diagnostic ultrasound system that can be widely used in hospitals at all levels.

## Materials and methods

### Subjects

This study was performed in the Department of Ultrasound Diagnosis of the Second Xiangya Hospital between May 2020 and October 2022. One hundred fifty-three patients underwent transrectal prostate examination, with a total of 153 suspicious prostate nodules. The average age of the patients was 66.78 ± 7.52 (47‒79). The mean PSA level was 22.23 ± 13.56 ng/mL (4.87‒69 ng/mL). The average prostate volume was 49.01 ± 13.62 mL (30.89‒81.02 mL). The inclusion criteria were as follows: 1) Patients with serum PSA levels ≥ 4.0 ng/mL or nodules detected by DRE; 2) Patients with no history of prostate surgery or chemoradiotherapy; 3) Patients with complete clinical data; and 4) Patients and their relatives who knew the precautions and risks related to ultrasound examination and needle biopsy. The exclusion criteria were as follows: 1) Acute and chronic urinary tract infections; 2) History of treatment for prostate lesions; 3) Allergy to ultrasound contrast agents and drugs; 4) Severe organ dysfunction; 5) Severe coagulation disorders; and 6) Cognitive dysfunction. This study followed the CONSORT Statement rules. All patients participating in this study signed informed consent forms, and this project was approved by the Ethics Committee of the Second Xiangya Hospital of Central South University [(2020) n° 072].

### Grayscale ultrasound examination

**Fig. 1:**
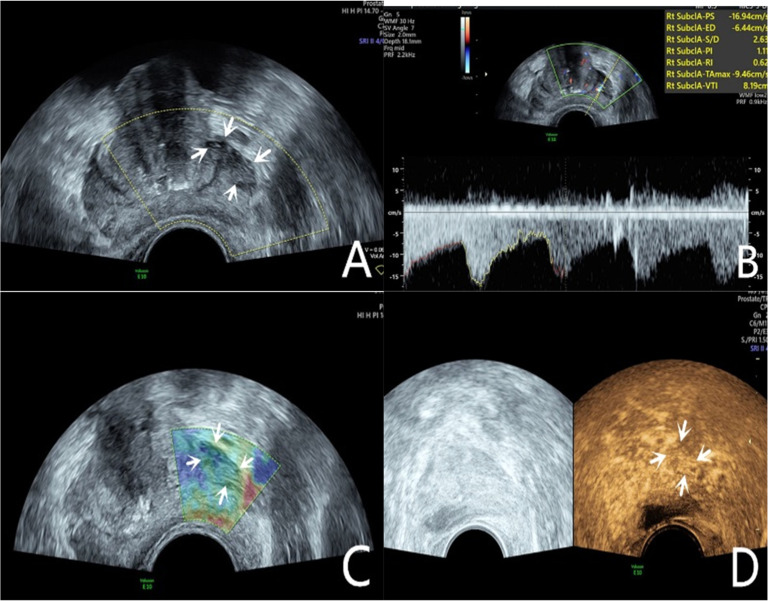
A 76-year-old patient had a total PSA of 14.3 ng/mL. Multiparameter US started with conventional transrectal ultrasound, and the lesion was a hypoechoic nodule at the junction of the inner and outer glands in the prostate’s left lobe (A, arrow). The lesion appeared on CDU with rich blood flow in the arterial spectrum (B, arrow). The operator used the endocavitary transducer to alternate between compressing and decompressing the lesion, which appeared mostly blue on TRTE (C, arrow). A hypoechoic nodule appeared on CEUS as a hypervascular nodule with a “fast in, fast out” enhancement pattern (D, arrow). Histopathology showed that the prostate lesions were clinically significant and Gleason 4 + 3 PCa.

**Fig. 2:**
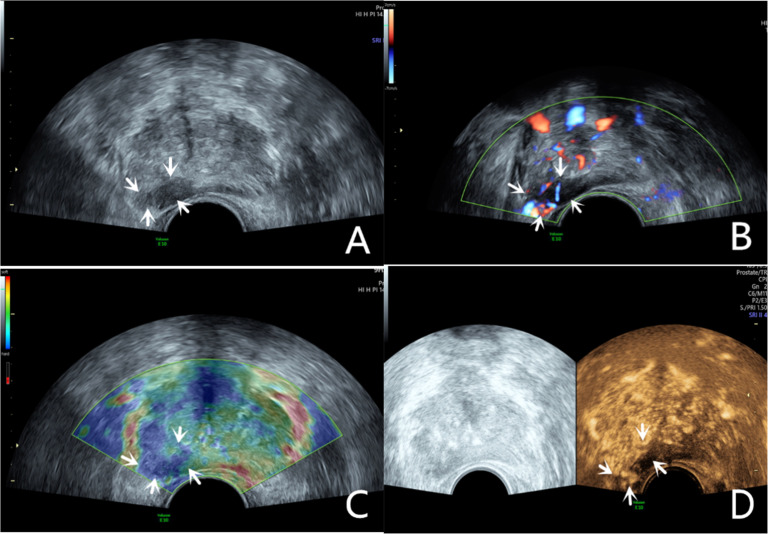
The lesion was a hypoechoic nodule in the outer glands of the prostate’s right lobe (A, arrow). The lesion appeared on CDU with rich blood flow (B). The operator used the endocavitary transducer to alternate between compressing and decompressing the lesion, which appeared mostly blue on TRTE (C). A hypoechoic nodule appeared on CEUS as a hypervascular nodule with hyperenhancing pattern (D).

Transrectal GSU was routinely performed using a GE Voluson E10 ultrasound system (GE Healthcare, Milwaukee, WI, USA) with a 3.5‒8 MHz rectal convex array probe (RIC 5–9-D). Before the examination, the patients were asked to empty their bowels and were positioned in a left lateral decubitus position. Then, a sonographer inserted the probe into the patient's rectum and preliminarily evaluated the shape, size, internal parenchymal echo, and integrity of the capsule of the prostate through multiple views, such as longitudinal and transverse sections. The criteria for GSU were as follows: a hypoechoic lesion in the prostate parenchyma was suspiciously positive, and isoechoic or hyperechoic lesions were considered negative (Fig. 1A; Fig. 2A).^10^

### Doppler ultrasound examination

The CDU/PDU examination of suspicious areas followed the grayscale ultrasound examination described above. The richness of blood flow in the parenchyma and the presence or absence of abnormal blood flow branches were observed. The criteria for Doppler ultrasonography were as follows: areas with rich blood flow or multiple abnormal blood flow branches compared with the surrounding normal prostate tissue were suspiciously positive, and areas with consistent blood flow or reduced blood flow compared to the surrounding normal tissue were considered negative (Fig. 1B; Fig. 2B).^14^

### Transrectal real-time tissue elastography

By engaging the dual display function for grayscale images and elastic images at the same time, TRTE examination on the hypoechoic area from GSU and the area with abnormally increased blood flow from Doppler ultrasound was performed. The suspicious area was placed in the sampling frame, and the probe was used to regularly and gently press the gland 1‒2 times/second with a pressure index of approximately 4. The optimal force and frequency of the manual compressions of the prostate were monitored by the visual color bar and waveform indicators. The images were frozen and stored after 3‒5 stable waveforms appeared in the elastogram. Different colors on the TRTE elastogram marked different densities of prostate tissue, with blue indicating hard tissues and red indicating soft tissues. PCa is stiffer than normal prostate tissue and often appears blue on TRTE images due to increased cell density, microvascularization, and collagen deposition from the matrix reaction. The criteria for TRTE were as follows: a blue area > 50 % in the nodule was suspiciously positive, and an area ≤ 50 % was considered negative (Fig. 1C; Fig. 2C).^15^

### Contrast-enhanced ultrasound examination

The microbubble suspension-based contrast agent SonoVue (Bracco Imaging SpA, Milano, Italy, 2.4 mL) was fully mixed with 5 mL of 0.9 % NaCl solution and was administered intravenously. The examination was started after the injection of the microbubble suspension and performed with a mechanical index of 0.2. Data such as contrast enhancement time, enhancement intensity, and contrast agent disappearance time in the suspiciously marked lesions of the prostate were recorded synchronously. After the contrast agent completely dissipated, the sonographer repeated the same examination again. The criteria for CEUS were as follows: early hyper enhancing areas in the prostate were suspiciously positive, and areas of simultaneous or late, isoenhancing or hypo-enhancing areas were considered negative (Fig. 1D; Fig. 2D).^16^ The results of all the above examinations were judged independently by two sonographers. Both sonographers are professors of ultrasonic diagnostics. The data used for the later logistic regression analysis were the results from only a senior experienced sonographer. The data for the repeatability test were additionally obtained from another sonographer with similar clinical experience who examined a random sample of patients compared to the previous sonographer.

### Needle biopsy and histological examination

After excluding patients with coagulation disorders, and urinary tract infections before radical prostatectomy, TRUS-guided 12 core systematic needle biopsies and two additional biopsies into suspicious lesions were performed. Twelve biopsy cores were taken routinely, including 3 samples from the peripheral zone of the prostate and 3 samples from the inner gland on each side. Most patients with suspected PCa who underwent TRUS biopsy underwent MRI. Therefore, the authors use the cognitive fusion of MRI/TRUS, which is also the most common and economical method in clinical practice. The puncture points for targeted biopsy were hypoechoic areas on GSU, areas with rich blood flow on Doppler ultrasound, stiff tissue on TRTE, areas with early hyperenhancement on CEUS, and suspected areas on MRI. The punctured specimens were placed in a 4 % formaldehyde solution and sent to the pathology department for diagnosis. All biopsy specimens were numbered to match the focal lesions, and the pathologic standard was based on the biopsy core. The histopathologic analysis was performed by an experienced pathologist, who was blinded by the US suspicion. The Gleason score for each core was recorded. The details are provided in Table 1.

**Table 1:** Ultrasonographic assignment for prostate nodules.

Observed indicators	Assignment	
	Negative	Positive
Echoes on GSU	Isoechoic or hyperechoic lesions	Hypoechoic lesion
Blood flow on CDU/PDU	Areas with consistent blood flow or reduced blood flow compared to the surrounding normal tissue	Areas with rich blood flow or multiple abnormal blood flow branches
Enhancement strength on CEUS	Iso-enhancing or hypo-enhancing areas	Hyper-enhancing areas
Time to enhancement on CEUS	Simultaneous enhancement or late enhancement	Early enhancement
Presence of a blue area on TRTE	≤ 50 %	> 50 %

GSU, Grayscale Ultrasound; CDU/PDU, Color Doppler Ultrasound/Power Doppler Ultrasound; TRTE, Transrectal Real-Time Tissue Elastography; CEUS, Contrast-Enhanced Ultrasound.

### Statistical analysis

SPSS 18.0 (IBM, Armonk, NY, USA) software and MedCalc (9.2.0.0, Broekstraat, Mariakerke, Belgium) software were employed for statistical analysis of the data. Continuous variables are presented as the mean ± standard deviation, categorical variables are represented as frequencies, and count data are illustrated as n (%). The chi-square test was used for comparisons between groups. Receiver Operating Characteristic (ROC) curves were drawn, and the Areas Under the Curve (AUCs) were calculated to evaluate the diagnostic effectiveness of each ultrasonic modality alone and in combination. Delong analysis was used to compare differences in diagnostic effectiveness among modalities. Ultrasonographic manifestations of prostate nodules were used as independent variables, with pathological results of biopsy as a dependent variable, in a logistic regression equation to fit a multivariate logistic regression model combining four ultrasonic methods. Krippendorff's alpha test was used to evaluate agreement between two sonographers. The reliability value was closer to 1 and suggested better agreement. A p-value of <0.05 indicated that the difference was statistically significant.

## Results

A total of 153 suspicious prostate lesions underwent needle biopsy, and 81 lesions were diagnosed as PCa (18 with Gleason scores ≤ 6, 29 with Gleason scores 7, and 34 with Gleason scores ≥ 8); 53 lesions were benign prostatic hyperplasia, and 19 lesions were hyperplasia with inflammation.

### Comparison of pathological results and diagnostic performances of GSU, CDU/PDU, TRTE and CEUS

GSU examined 153 lesions and found 102 hypoechoic areas, 31 isoechoic areas, 12 hyperechoic areas, and 8 anechoic areas. Compared with the pathological results, GSU had a Sensitivity (SE), Specificity (SP), Positive Predictive Value (PPV), Negative Predictive Value (NPV), and Accuracy Rate (AR) for diagnosing PCa of 70.4 %, 37.5 %, 55.9 %, 52.9 %, and 54.9 %, respectively. CDU/PDU examined 153 lesions. The results showed that 100 lesions had rich blood flow, and no obvious increased blood flow or branches with abnormal blood flow were found in 53 lesions. Compared with the pathological results, CDU/PDU had a SE, SP, PPV, NPV, and AR for diagnosing PCa of 69.1 %, 38.9 %, 56.0 %, 52.8 %, and 54.9 %, respectively. TRTE examined 153 lesions and found 87 positive lesions and 66 negative lesions. Compared with the pathological results, TRTE had a SE, SP, PPV, NPV, and AR for diagnosing PCa of 90.1 %, 80.6 %, 83.9 %, 87.9 %, and 85.6 %, respectively. CEUS examined 153 lesions and found 93 hyper-enhancing lesions, 37 isoenhancing lesions, 13 mildly enhancing lesions, and 10 non-enhancing lesions. Compared with the pathological results, CEUS had a SE, SP, PPV, NPV, and AR for diagnosing PCa of 79.0 %, 59.7 %, 68.8 %, 71.7 %, and 69.9 %, respectively. The details are provided in Table 2, Table 3.

**Table 2:** The performances of GSU, CDU/PDU, TRTE, CEUS for diagnosing PCa (number).

Pathological	GSU			CDU/PDU			TRTE			CEUS		
	Positive	Negative	Sum	Positive	Negative	Sum	Positive	Negative	Sum	Positive	Negative	Sum
Positive	57	24	81	56	25	81	73	8	81	64	17	81
Negative	45	27	72	44	28	72	14	58	72	29	43	72
Sum	102	51		100	53		87	66		93	60	

GSU, Grayscale Ultrasound; CDU/PDU, Color Doppler Ultrasound/Power Doppler Ultrasound; TRTE, Transrectal Real-Time Tissue Elastography; CEUS, Contrast-Enhanced Ultrasound.

**Table 3:** ROC curve analysis of multiple modalities in benign and malignant prostate lesions.

Modalities	SE (%)	SP (%)	PPV (%)	NPV (%)	AR (%)
GSU	70.4	37.5	55.9	52.9	54.9
CDU/PDU	69.1	38.9	56.0	52.8	54.9
TRTE	90.1	80.6	83.9	87.9	85.6
CEUS	79.0	59.7	68.8	71.7	69.9
χ^2^	4.62^a^	10.6^a^	6.26^a^	8.0^a^	7.46^a^
p	0.032^a^	0.001^a^	0.012^a^	0.005^a^	0.006^a^

GSU, Grayscale Ultrasound; CDU/PDU, Color Doppler Ultrasound/Power Doppler Ultrasound; TRTE, Transrectal Real-Time Tissue Elastography; CEUS, Contrast-Enhanced Ultrasound; SE, Sensitivity; SP, Specificity; PPV, Positive Predictive Value; NPV, Negative Predictive Value; AR, Accuracy Rate.

a The results of the chi-square test between TRTE and CEUS.

### Comparison of the diagnostic efficacies of different ultrasonic modalities

**Fig. 3:**
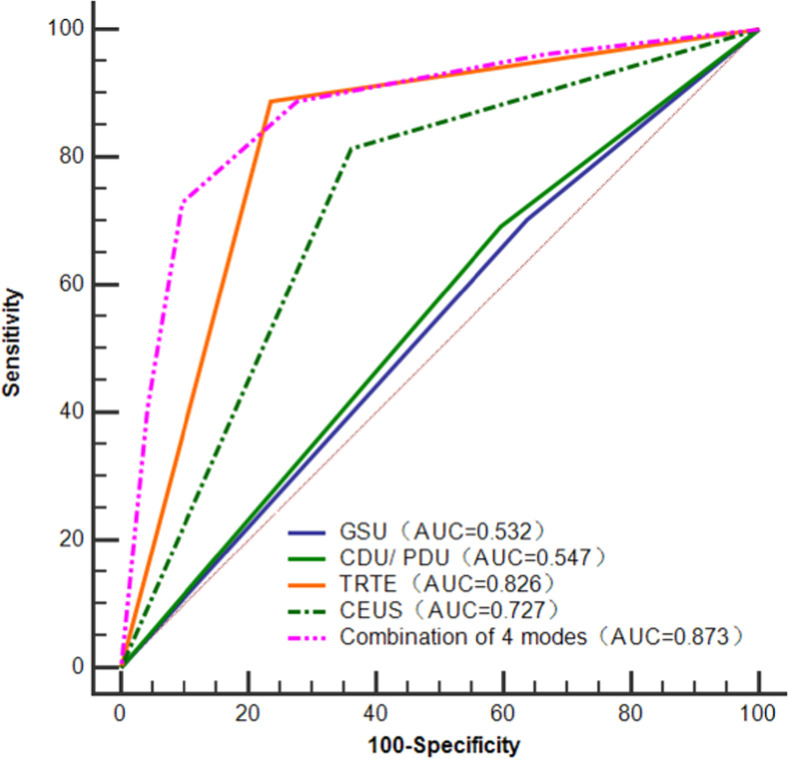
ROC curves of GSU, CDU/PDU, TRTE, CEUS and their combination for diagnosing prostate lesions.

According to the results of the chi-square test, the SE, SP, PPV, NPV, and AR for PCa detection of TRTE were higher than those of CEUS, and the differences were statistically significant (*p* < 0.05). Delong analysis showed that the AUC of TRTE was higher than that of CEUS (0.826 vs. 0.727, *p* = 0.046). Moreover, the results of the ROC curve analysis showed that the diagnostic performance of the combination of the four models for diagnosing PCa had an AUC = 0.873 (95 % CI 0.810, 0.922), which was significantly higher than that of either methods GSU = 0.532 (0.450, 0.613), CDU/PDU = 0.547 (0.465, 0.628), TRTE = 0.826 (0.757, 0.883), CEUS = 0.727 (0.649, 0.796)) (Fig. 3).

### The results of the logistic regression analysis

The ROC curves of patient age, prostate volume, and serum total PSA were drawn, and the optimal cut-off points were 66 years old, 49.42 mL, and 25.64 ng/mL, respectively. Taking patient age, prostate volume, serum total PSA, and the performances of the four ultrasonic methods for diagnosing prostate nodules as independent variables and the pathological results as the dependent variable, the results of binomial logistic regression analysis showed that enhancement strength on CEUS, time to enhancement on CEUS, presence of a blue area on TRTE and total serum PSA were statistically significant independent factors for diagnosing PCa. Multivariate logistic regression analysis was performed on these 4 independent influencing factors, and the results showed that enhancement strength on CEUS, presence of a blue area on TRTE, and total serum PSA were risk factors for PCa, as shown in Table 4.

**Table 4:** The results of binomial and multivariate logistic regression analysis.

Observed indicators	Binomial logistic regression analysis			Multivariate logistic regression analysis		
	OR	95 %CI	p	OR	95 %CI	p
Echoes on GSU	1.34	0.68‒2.64	0.39	‒	‒	‒
Blood flow on CDU/PDU	1.51	0.78‒2.94	0.23	‒	‒	‒
Enhancement strength on CEUS	5.77	2.86‒11.67	<0.01	3.38	1.18‒9.70	0.02
Time to enhancement on CEUS	4.30	2.18‒8.48	0.01	2.59	0.87‒7.69	0.09
Presence of a blue area on TRTE	4.76	2.39‒9.48	<0.01	6.61	2.77‒15.81	<0.01
Patient's age (cut-off value was 66-years-old)	1.62	0.79‒3.30	0.19	‒	‒	‒
Prostate volume (cut-off value was 49.42 mL)	0.55	0.28‒1.09	0.09	‒	‒	‒
Total serum PSA (cut off value was 25.64 ng/mL)	4.60	2.27‒9.34	<0.01	3.13	1.38‒7.11	0.01

GSU, Grayscale Ultrasound; CDU/PDU, Color Doppler Ultrasound/Power Doppler Ultrasound; TRTE, Transrectal Real-Time Tissue Elastography, CEUS, Contrast-Enhanced Ultrasound, PSA, Prostate Specific Antigen, OR, Odds Ratio.

The results of the repeatability test showed that the agreement between the two sonographers was 0.76 (95 % CI 0.71‒0.80) for GSU, 0.73 (95 % CI 0.69‒0.78) for CDU/PDU, 0.78 (95 % CI 0.73‒0.82) for TRTE, and 0.73 (95 % CI 0.67‒0.78) for CEUS.

## Discussion

Currently, conventional GSU is used for detecting PCa, and guiding systematic biopsy and the placement of radiotherapy particles. Approximately 60 % of PCa lesions on GSU are hypoechoic,^10^ and approximately 35 %‒39 % are isoechoic^17^ and hyperecho.^18^ In clinical practice, GSU imaging has limitations in PCa detection as the backscatter signals from PCa, and normal prostate can be similar. GSU has SE in PCa detection ranging only from 11 % to 35 % and a positive predictive range from 17 % to 57 %.^19^ Moreover, a study by Klein et al. showed that GSU has a poor SP for early PCa, with a false-negative rate of up to 30 % for the pathological results of systematic biopsy guided by GSU.^20^ In this study, the authors also found that the diagnostic performance and sensitivity of GSU for detecting PCa were not very excellent.

The normal prostate is an organ with a low blood supply. However, hyperplasia, inflammation or malignant nodules in the prostate increase blood flow. Therefore, CDU and PDU are commonly used clinically to detect abnormal proliferative blood vessels in the prostate and identify malignant lesions that are not visible on GSU. The SE for PCa detection increases when CDU is utilized, improving the diagnostic performance of GSU. PDU is more sensitive in detecting microvascular perfusion than CDU. In a study of 620 patients before radical prostatectomy, Eisenberg ML et al. found that the additional use of PDU with GSU could increase the SP from 47 % to 74 %.^21^ Although PDU is more sensitive than CDU in detecting slow blood flow, PDU has not shown better PCa detection than CDU.^22^ In addition, PDU does not depict the direction of the blood flow. The results of the present study showed that the SE, SP, PPV, NPV, and AR of CDU/PDU in diagnosing PCa were similar to those of GSU. Therefore, in clinical practice, the diagnostic performance of PCa by Doppler ultrasound is not very ideal. The major limitations of CDU are operator dependency and lack of standardization. Thus, Doppler ultrasonography can only be used as a primary screening method for PCa. CDU and PDU can only present intralesional vessels with diameters greater than 100 µm, and neither is sufficient in detecting early PCa. CEUS is a more easily visualized microvessel with an internal diameter of 10‒50 µm.

Prostatic adenocarcinoma is characterized by angiogenesis with increases in microvasculature density. A large number of microvessels generated in PCa provide nutrients for proliferation, metastasis, and invasion of tumors, and the Microvessel Density (MVD) of PCa is significantly higher than that of normal prostate tissue. MVD is an index reflecting the degree of neovascularization in the tumor. The degree of MVD increases with the Gleason score of PCa.^13^ In a prospective study of 65 patients with elevated PSA, Zhao et al. found that the SE and SP of CEUS for the diagnosis of PCa were 79.3 % and 86.1 %, respectively.^23^ In a meta-analysis of 16 studies with a total of 2624 patients, Li et al. found that the SE and SP of CEUS imaging in detecting prostate cancer were 0.70 and 0.74, respectively.^24^ These results showed that CEUS has a good diagnostic performance for PCa, similar to the results of this study. The authors found that CEUS had satisfactory diagnostic performance in diagnosing PCa. In clinical biopsy, several studies have reported that the detection rate of PCa in CEUS-guided targeted biopsy is 10.4 %‒32 %, which is higher than that of systematic biopsy (5.2 %‒18 %).^24^^,^^25^ CEUS brings several advantages in the management of PCa including diagnosis, facilitating targeted prostate biopsy, real-time evaluation, and identification of post-treatment recurrence. However, CEUS has a high SE for the diagnosis of larger tumors located in the peripheral zone of the prostate and underdiagnoses tumors in the transition zone.^26^ In addition, PCa nodules that are small without a formed neovascular network have no characteristic CEUS manifestations.

Elastography has been used clinically to examine various organs, including the breast, thyroid, and prostate, since its first application by Ophir et al.^27^ 85 % of PCa is multifocal and progresses along the capsule of the prostate, and it may be nodules with blurred borders.^28^ Therefore, it is difficult for conventional imaging techniques to accurately detect lesions. TRTE is another diagnostic ultrasonic technique that can assess the stiffness of prostate lesions. Several studies have reported the important value of TRTE in the diagnosis of PCa. Zhang B et al. reported that the total SE and SP of TRTE for the diagnosis of PCa were 0.72 and 0.76, respectively.^12^ The research of Ding et al.^29^^,^^30^ showed that the SE, SP and AUC of TRTE in diagnosing PCa were 0.835, 0.844 and 0.870, respectively. The PCa detection rate of TRTE-guided targeted biopsy was 4.7 times higher than that of systematic biopsy.^31^ Moreover, the detection rate of TRTE for PCa is comparable to that of mpMRI.^32^ The results of the present study showed that the diagnostic performance of TRTE for PCa was better than that of CEUS. This is similar to the results reported in several studies. Jieun Koh et al.^33^ prospectively analyzed 52 patients with suspected PCa, and the positive rate of targeted biopsy in the TRTE group was higher than that in the CEUS group (*p* < 0.05). Adding TRTE to the CEUS examination before radical prostatectomy can improve the detection rate of PCa. A review of the application of multiple ultrasound modalities in diagnosing PCa showed that elastography demonstrated a better diagnostic performance than CEUS and was more suitable for clinical practice.^34^ TRTE has the advantage of detecting lesions in the top and middle of the prostate, and it presents ideal diagnostic performance in PCa diagnosis.

The purpose of this study was to evaluate the performance of multiple ultrasonic modalities in detecting PCa, and the results showed that the combined diagnostic accuracy of GSU, CDU/PDU, TRTE, and CEUS for PCa was significantly higher than that of any modality alone. Among the current diagnostic imaging studies for PCa, few have investigated multiparametric Ultrasound (mpUS) combined with multiple ultrasonic modalities. Zhang et al. performed mpUS and mpMRI in all of their 88 patients, and the results showed that the SE, NPV, and AUC for detecting PCa were higher for mpUS than for mpMRI (97.4 % and 94.7 %, 96.9 % and 92.3 %, 0.874 and 0.774, respectively).^35^ Wildeboer et al. combined B-mode, shear-wave elastography, and dynamic CEUS for the diagnosis of PCa, the results concluded the feasibility of a multiparametric classifier to improve upon single modalities for the detection of PCa.^36^ The combination of acoustic radiation force impulse, shear wave elasticity imaging, quantitative ultrasound, and B-mode significantly improved the diagnostic efficiency of PCa.^37^ The above studies show the good performance of mpUS in diagnosing PCa. The authors found that the clinical implementation of mpUS was safe and this process could be completed within 30 min with good imaging quality. Therefore, the combined application of multiple ultrasonic modes in this study can assist clinical diagnosis of PCa and assess its malignancy, which can detect high-risk PCa to a greater extent and detect low-risk PCa to a lesser extent to avoid overtreatment.

The present study had some limitations. Firstly, the current study is represented by its retrospective design on an operator-dependent technology. Secondly, PCa is diagnosed using TRUS-guided needle core biopsies rather than thin-section, whole-mount prostatectomy specimens.

In conclusion, the diagnostic performance of TRTE for PCa is better than that of CEUS. Early hyperenhancement on CEUS and the presence of a blue area on TRTE have significant diagnostic value for PCa. Moreover, compared with a single ultrasonic technology, the combination of multiple ultrasonic technologies can significantly improve the positive rate of PCa.

## Guarantor

The scientific guarantor of this publication is Ran Xu.

## Informed consent

All patients participating in this study signed informed consent forms.

## Ethical statement and consent to participate

All procedures performed in studies involving human participants were in accordance with the ethical standards of the institutional and/or national research committee [(2020) No. 072].

## Consent for publication

Not applicable.

## Funding

This study was supported by the 10.13039/501100001809National Natural Science Foundation of China (n° 81871372), 10.13039/501100004735Natural Science Foundation of Hunan Province (n° 2023JJ30743), and Natural Science Foundation of Changsha (kq2208341).

## CRediT authorship contribution statement

**Yushan Liu:** Conceptualization, Investigation, Methodology, Data curation, Writing – original draft. **Shi Zeng:** Funding acquisition, Resources. **Dan Zhou:** Software, Supervision, Validation, Visualization. **Haiqing He:** Software, Supervision, Validation, Visualization. **Shuiqing Wu:** Software, Supervision, Validation, Visualization. **Changkun Huang:** Software, Supervision, Validation, Visualization. **Kai Ai:** Software, Supervision, Validation, Visualization. **Xuan Zhu:** Software, Supervision, Validation, Visualization. **Ran Xu:** Formal analysis, Project administration, Writing – review & editing.

## Declaration of competing interest

The authors declare no conflicts of interest.
